# Concomitant immunoglobulin A nephropathy and membranous nephropathy with Fabry-like zebra bodies: A case report and literature review

**DOI:** 10.1097/MD.0000000000047912

**Published:** 2026-02-28

**Authors:** Yujie Dai, Chunlei Lu, Guangyu Bi, Gang Zhou, Rong Wang

**Affiliations:** a Ruikang Clinical Medical College, Affiliated to Guangxi University of Chinese Medicine, Nanning, China; b Department of Nephrology, Northern Jiangsu People’s Hospital, Yangzhou, China.

**Keywords:** Fabry disease, immunoglobulin A nephropathy, membranous nephropathy

## Abstract

**Rationale:**

Immunoglobulin A nephropathy (IgAN) and membranous nephropathy (MN) are common primary glomerular diseases. Although these conditions are well-characterized, their co-occurrence with Fabry-like zebra bodies is exceptionally rare. Fabry-like zebra bodies, characterized pathologically by numerous myeloid bodies within glomerular podocytes, are a hallmark feature of classical Fabry disease. However, similar ultrastructural findings can also arise from other genetic disorders or drug-induced phospholipidosis (e.g., associated with hydroxychloroquine or amiodarone).

**Patient concerns:**

The patient was a 51-year-old woman with clinical manifestations dominated by massive proteinuria and normal creatinine levels.

**Diagnoses:**

The patient was diagnosed with IgAN and MN with Fabry-like zebra bodies.

**Interventions:**

Treatment comprised angiotensin-converting enzyme inhibitors and angiotensin II receptor blockers.

**Outcomes:**

After 1.5 years, she exhibited considerable decreases in creatinine from 61 μmol/L to 42.9 μmol/L and proteinuria from 3.8 g/24 h to 52.5 mg/24 h. Overall, the patient’s clinical course was relatively smooth. Despite transient fluctuations in renal parameters, the patient achieved significant remission of proteinuria and edema following treatment.

**Lessons:**

This case highlights the complexity of diagnosing and managing the co-occurrence of IgAN, MN, and Fabry-like zebra bodies, emphasizing the need for further research on its pathogenesis, treatment, and prognosis.

## 1. Introduction

Immunoglobulin A nephropathy and membranous nephropathy (MN) are 2 distinct forms of glomerular diseases. Fabry-like zebra bodies refer to pathological or clinical findings resembling those seen in Fabry disease, but which occur in individuals without confirmed genetic mutations in galactosidase alpha. They are rare in patients with immunoglobulin A nephropathy (IgAN) and MN. Accurate diagnosis and appropriate treatment of these conditions require comprehensive renal pathological examination. Fabry disease (FD) is an X-linked, hereditary, lysosomal storage disease caused by deficiency of the enzyme α-galactosidase A, which results in the accumulation of the neutral glycosphingolipid (GSL) globotriaosylceramide (GL-3) in the walls of small blood vessels, nerves, dorsal root ganglia, renal glomerular and tubular epithelial cells, and cardiomyocytes. Fabry-like bodies are key pathological manifestations of this deposition in the renal tubules; however, they are not exclusively found in FD. We can also find them in podocyte, retinal epithelial cell, neuroepithelial cell, hepatic cell, and so on. Other diseases caused by reactions to drugs such as hydroxychloroquine and amiodarone, as well as other genetic diseases, can also cause Fabry-like changes.

Herein, we present a diagnostically challenging case of concurrent IgAN and MN accompanied by Fabry-like zebra bodies. This report highlights the exceptional rarity of this triple pathological combination and provides valuable insights into the complex interplay between different glomerular disease processes. Furthermore, it emphasizes the critical importance of developing individualized treatment strategies for patients with overlapping glomerular pathologies.

## 2. Case report

A 51-year-old woman presented with a 2-week history of symmetrical bilateral lower leg pitting edema. The results of laboratory tests are summarized in Table 1. Her 24-hour urine protein was 3.8 g, plasma albumin level was 24.2 g/L, and serum creatinine was 44 µmol/L. Tests for human immunodeficiency virus and syphilis were negative. Although she tested positive for the hepatitis B core antibody, the surface antigen was negative. Immunological tests, including immunofixation electrophoresis, anti-glomerular basement membrane antibodies, double-stranded DNA, antinuclear antibodies, and antineutrophil cytoplasmic antibodies, were all negative. Her serum immunoglobulin concentrations were as follows: elevated immunoglobulin G (IgG) at 4.89 g/L. The IgG kappa (κ) level was 1.46 mg/mL and IgG lambda (λ) level was 0.85 mg/mL, with an abnormal κ/λ ratio (κ/λ = 1.72). Ultrasonography revealed that both kidneys were normal in size (left kidney, 119 × 50 mm; right kidney, 105 × 57 mm) and morphology.

**Table 1:** Patient’s laboratory examination results.

Hemoglobin	151 g/L	Serum phospholipase A2 receptor	Negative
White blood cell count	6.31 × 10^9^/L	Hepatitis B core antibody	Positive
Blood platelet	283 × 10^9^/L	Antineutrophil cytoplasmic antibody	Negative
D-Dimer	0.66 µg/L	Anti-glomerular basement membrane antibody	Negative
Albumin	24.2 g/L	Antinuclear antibody	Negative
Total cholesterol	13.55 mg/dL	Immunoglobulin G	4.89 g/L
Triglycerides	1.91 mmol/L	Immunoglobulin A	1.6 g/L
Serum creatinine	44 µmol/L	Serum kappa light chain	1.46 mg/mL
24-hour urinary protein excretion	3.8 g/24 h	Serum lambda light chain	0.85 mg/mL
Urine red blood cell	105.8/µL	Kappa/lambda	1.72

**Figure 1.**
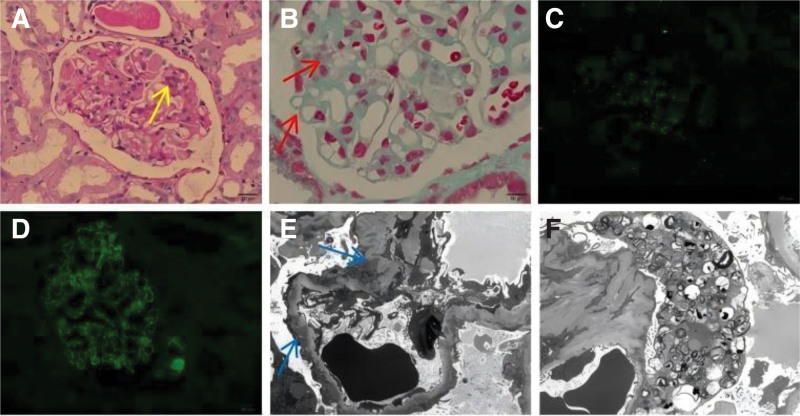
Renal biopsy results. (A) Light microscopy showing thickening of the glomerular basement membrane, with mesangial expansion and variable mesangial hypercellularity (yellow arrow) (×400 magnification). (B) Masson staining showed eosinophilic deposits in the subepithelial area and mesangial area (red arrow) (×1000 magnification). (C) IgA immunofluorescence showing IgA mesangial deposits (×400 magnification). (D) IgG immunofluorescence showing granular capillary wall staining for IgG (×400 magnification). (E) Electron microscopy image showing electron-dense deposits in the subepithelial space and mesangial area (blue arrow) (×5000 magnification). (F) Electron microscopy revealing myelin figures and zebra bodies in podocytes, and electron-dense materials in the mesangium (×5000 magnification). IgG = immunoglobulin G.

Renal biopsy was conducted (Fig. 1). Light microscopy revealed distinct pathological features, including diffuse thickening of the glomerular basement membrane accompanied by mesangial expansion and focal mesangial hypercellularity (Fig. 1A). Masson trichrome staining demonstrated granular eosinophilic deposits in both the subepithelial and mesangial areas (Fig. 1B). Immunofluorescence microscopy demonstrated prominent mesangial deposition of IgA (Fig. 1C), along with diffuse granular deposits of IgG and C3 distributed along the capillary walls (Fig. 1D). Immunofluorescence staining for the M-type phospholipase A2 receptor (PLA2R) was negative. Ultrastructural analysis by electron microscopy revealed multiple electron-dense deposits localized in the subepithelial space and intramembranous regions, associated with extensive effacement of podocyte foot processes (Fig. 1E). Sparse electron-dense deposits were identified in the mesangial matrix (Fig. 1E). Electron microscopy demonstrated numerous membrane-bound concentric lamellar structures consistent with myeloid bodies within glomerular podocytes, exhibiting extensive cytoplasmic fusion (Fig. 1F). Conversely, clinical evaluation revealed no characteristic features of FD.

The patient lacked any identifying symptoms of FD, such as the absence of corneal opacities; angiokeratoma; characteristic bizarre, intermittent, and transient acroparesthesia; gastrointestinal symptoms (postprandial cramps, diarrhea, or achalasia); peripheral neuropathy; or cerebrovascular involvement. Cardiac assessment, including two-dimensional echocardiography, demonstrated normal cardiac structure and function, with normal dimensions of both the ventricles and atria, and preserved systolic and diastolic functions. Pulmonary evaluation, including chest radiography and pulmonary function tests, yielded normal results. Definitive diagnostic testing, including measurement of α-galactosidase A activity and genetic analysis, revealed no abnormalities indicative of FD.

Based on a comprehensive analysis of pathological findings and clinical characteristics, the patient was diagnosed with concurrent IgAN and MN accompanied by Fabry-like zebra bodies. The therapeutic regimen primarily comprised angiotensin-converting enzyme inhibitors (ACEIs) and angiotensin II receptor blockers (ARBs). During the 18-month follow-up period, the patient demonstrated a significant clinical decrease in creatinine from 61 μmol/L to 42.9 μmol/L, and in proteinuria from 3.8 g/24 h to 52.5 mg/24 h, with a marked resolution of proteinuria and amelioration of associated symptoms such as edema.

## 3. Discussion

Herein, we report the case of a female patient with coexisting IgAN and MN accompanied by Fabry-like zebra bodies and symptoms of massive proteinuria who received treatment with ACEIs or ARBs for 1.5 years, achieving complete remission (CR). The rarity of this case encourages the exploration of novel biomarkers or therapeutic targets for similar disorders, and underscores the need for a thorough differential diagnosis to avoid misdirected therapies and ensure appropriate management of the true underlying condition.

IgAN is one of the most common types of glomerulonephritis, and is characterized by the deposition of IgA in the glomerular mesangium.^[1]^ Comprehensive laboratory and imaging investigations are essential to exclude secondary etiologies, including hepatic diseases, malignancies, autoimmune disorders (e.g., systemic lupus erythematosus and rheumatoid arthritis), and chronic infections.

MN is a common cause of nephrotic syndrome in adults.^[2]^ Pathologically, deposits at sites other than the subepithelial aspect of the glomerular basement membrane favor the presence of secondary forms of membranous glomerulopathy which are seen most commonly in the setting of autoimmune disease, infection, neoplasia, and with certain therapeutic agents.^[3]^ Primary MN is characterized by IgG4-rich deposits, sometimes including IgG1, particularly in very early disease.^[4,5]^ PLA2R is a transmembrane protein expressed in podocytes. The majority of cases of primary MN are associated with auto-antibodies against the podocyte antigen-PLA2R.^[6]^ Newly discovered target antigens have addressed this knowledge gap in PLA2R-negative MN, with genes including Neural Epidermal Growth Factor-Like Protein 1, Exostosin 1 and Exostosin 2, and Neural Cell Adhesion Molecule 1.^[2]^ In the present case, the absence of hepatitis B viremia despite isolated core antibody positivity supported the diagnosis of idiopathic MN. Immunofluorescence microscopy revealed glomeruli with marked staining of the mesangial areas for IgA and subepithelial regions for IgG. Therefore, histopathological evaluation confirmed concurrent primary IgAN and MN, based on the exclusion of secondary causes.

There have been a few rare reports on the concurrence of IgAN and MN. A search of the Pubmed database using the keywords “IgAN” and “MN” revealed 5 related reports, encompassing 40 patients. Of these 40 patients (mean age: 41 years), 17 underwent accurate treatment and follow-up. The male:female ratio was 3:2. Clinical manifestations were mainly characterized by varying degrees of proteinuria and hematuria. The average 24-hour urinary protein excretion was 4.7 g/24 h, and serum creatinine levels were mostly within the normal range. We subsequently focused on these patients and performed a meta-analysis to identify the factors of treatment interventions that could be significantly associated with diffferent prognosis or the incidence. We organized these cases into 2 categories: effective treatment, including partial remission, CR, or stable decreased proteinuria, and poor prognosis, which was defined as the occurrence of stable persistent proteinuria or all-cause mortality. Although longitudinal comparisons of proportions across different studies may be subject to bias owing to variations in the follow-up durations, cross-sectional comparisons within the same cohort revealed clinically relevant therapeutic interventions. The Chi-squared test revealed that treatment with ACEIs/ARBs was significantly associated with a good prognosis of partial remission, CR, or stable decreased proteinuria. Notably, corticosteroid pulse therapy and immunosuppressant therapy may also reduce proteinuria. Kobayashi et al^[7]^ previously suggested that IgAN and MN should be considered as entities of glomerular pathology. This may be due to different nonspecific antigens stimulating the body to produce an immune response 1 or more times. Alternatively, it may involve the same antigen simultaneously triggering a variety of antibody responses to form different immune complexes. There is no evidence to support a causal relationship between these 2 diseases in terms of their pathogenesis. According to some studies, patients with combined IgAN and MN display clinical features similar to those of patients with MN, but milder pathological lesions than those of patients with IgAN. Current evidence suggests favorable treatment responses to corticosteroid-based immunosuppression, with combination regimens incorporating cyclophosphamide demonstrating particular efficacy. Importantly, available studies indicate no synergistic adverse prognostic impact when these entities coexist.

In this patient, electron microscopy revealed extensive myeloid bodies restricted to the podocytes, unaccompanied by the characteristic extrarenal manifestations of FD, such as angiokeratomas, corneal opacities, or neuropathic pain. An unremarkable family history, normal α-galactosidase A activity, and exclusion of chloroquine exposure effectively ruled out classical FD, a multisystemic X-linked lysosomal storage disease caused by decreased alpha-galactosidase A activity that results in the lysosomal accumulation of neutral GSLs and GL-3.^[8]^

Fabry-like zebra bodies are rarely encountered in clinical practice. Genetic testing and enzyme activity assays should be considered for a comprehensive examination and differential diagnosis to exclude FD and support Fabry-like zebra bodies. Missed diagnoses and misdiagnoses can easily occur, complicating accurate judgment. Emerging evidence suggests that Fabry-like ultrastructural changes reflect lysosomal processing defects due to diverse etiologies,^[9]^ including acquired sphingolipid metabolism disturbances, medication effects, and novel genetic or epigenetic anomalies that affect lysosomal enzymes. Ultrastructural investigations have revealed that these cytosolic inclusions consisted of concentric myelin-like structures, the so-called lamellar bodies, the presence of which became the morphological hallmark of phospholipidosis.^[10]^ Lysosomes are involved in phospholipidosis. Classic lysosomal storage disorders are monogenic, and cause the accumulation of lysosomal substrates due to defective enzymes or proteins. Mutations in genes that encode enzymes can lead to various deficiencies, including undue posttranslational modifications.^[9]^ Previous case reports have been published that have drawn attention to chloroquine, which causes histomorphological changes similar to those in FD.^[9]^ It may also arise from secondary causes, such as acquired conditions (e.g., chronic kidney disease and diabetes), causing similar vascular or lipid deposition patterns.

Fabry-like zebra bodies are morphological features that represent only pathological changes. The presence of zebra body-like changes is the key pathological basis for the diagnosis of renal FD, including phospholipidosis. Although Fabry-like zebra bodies are the hallmark of FD, similar layered inclusions may occasionally occur in other conditions, a phenomenon known as “zebra body-like changes.” Such conditions are caused by other genetic diseases and also by reactions to drugs, such as hydroxychloroquine and amiodarone. FD is caused by an X-linked innate error in the GSL metabolic pathway that results in the lysosomal accumulation of GL-3 in a wide variety of cells, leading to various disease manifestations. Renal involvement can be confirmed by electron microscopy, which classically shows deposits of GL-3 that appear primarily as lamellated membrane structures called myeloid or zebra bodies that occur within enlarged secondary lysosomes. These findings are typically diffuse and consistent.^[11]^

There is currently no single, highly specific symptom that can directly indicate Fabry-like zebra bodies, and further exploration is needed. Further, the pathogenesis underlying the link between IgAN, MN, and Fabry-like zebra bodies remains unclear, although hypotheses have been proposed. Firstly, the deposition of immune complexes is involved in both IgAN and MN; this process may lead to glomerular damage and podocyte lesions, thereby inducing the formation of Fabry-like zebra bodies. Second, immune complex deposition may activate the complement system or inflammatory response, further exacerbating glomerular and podocyte injuries. In addition, abnormal metabolites (such as those associated with lipid metabolism disorders) may lead to lipid deposition in podocytes, forming Fabry-like zebra bodies. Moreover, long-term proteinuria or chronic kidney disease results in podocyte injury and lysosomal dysfunction, leading to pathological manifestations. Although nongenetic mutations in FD were found in this case, the possibility of other gene mutations or epigenetic changes could not be completely ruled out. These factors may act in conjunction with immune complex deposition, resulting in pathological changes. A Chinese report on the occurrence of IgAN with Fabry-like zebra bodies suggested that treatment with ACEIs or ARBs, along with immunosuppressants, may contribute to a significant and sustained decrease in 24-hour urine protein to a low level. Renal function can also improve and remain stable. Notably, our patient achieved proteinuria remission with ACEI/ARB therapy alone for >18 months. This outcome supports 2 critical observations. Firstly, Fabry-like zebra bodies in non-FD contexts may represent epiphenomena, rather than primary pathologies. Second, conservative management is sufficient for selected patients without progressive renal impairment.

In conclusion, this report discusses the clinical implications and potential therapeutic strategies for patients with IgAN, MN, and Fabry-like zebra bodies, and underscores the necessity for further research into the mechanisms underlying such combined glomerular injuries. The coexistence of IgAN, MN, and Fabry-like zebra bodies suggests that glomerular disease may not only result from a single pathological mechanism, but may rather stem from a combination of multiple factors. Therefore, clinicians must comprehensively consider the various possible causes, and provide individualized treatment plans for patients with overlapping renal pathologies. When medullary (zebra) bodies are detected by renal biopsy electron microscopy, the first step is to ensure that the pathology report accurately describes their morphological features and distribution, particularly their localization in podocytes. Zebra bodies are indicative of a lysosomal storage disease, but are not specific to FD. As such, patients presenting with this finding require a comprehensive evaluation through clinical history, medication history, and laboratory tests. Detailed inquiries should include family history (especially of early onset kidney disease, heart disease, or stroke in male relatives) and personal history (including acral pain, hypohidrosis, cutaneous angiofibromas, corneal opacity, gastrointestinal discomfort, hearing loss, or cardiovascular events). Particular attention should be paid to the long-term use of drugs such as chloroquine, hydroxychloroquine, amiodarone, and tamoxifen, which may cause drug-induced phospholipid deposition. For male patients, alpha-galactosidase A activity testing is recommended as significantly reduced or absent activity has diagnostic significance. All patients, particularly females, should undergo galactosidase alpha gene sequencing to confirm or exclude FD. If no enzymatic or genetic evidence exists, and there is no history of pathogenic drugs, other lysosomal storage diseases should be considered alongside clinical manifestations with corresponding enzymatic or genetic testing. If drug-related changes are strongly suspected, the suspected medication may need to be discontinued under medical supervision after weighing the risks, after which renal function monitoring and electron microscopy should be conducted. Overall, zebra bodies serve as important pathological clues; however, definitive diagnosis requires the comprehensive evaluation of each patient’s clinical presentation, medication history, enzymatic activity, genetic factors, and ultrastructural features to avoid misdiagnosis of other metabolic or drug-induced conditions.

Overall, this case highlights the complexity of IgAN, MN, and Fabry-like zebra bodies, and calls for increased vigilance to facilitate early diagnosis and appropriate treatment, which would minimize the morbidity associated with complex nephropathy. Further research should elucidate the molecular pathways linking immune complex deposition with lysosomal dysfunction and potentially identify novel therapeutic targets for multifaceted glomerulopathies.

## Author contributions

**Funding acquisition:** Rong Wang.

**Methodology:** Gang Zhou.

**Project administration:** Guangyu Bi, Rong Wang.

**Resources:** Guangyu Bi.

**Software:** Gang Zhou.

**Supervision:** Rong Wang.

**Writing – original draft:** Yujie Dai.

**Writing – review & editing:** Chunlei Lu.
